# Identifying hypertensive disorders of pregnancy, a comparison of two epidemiologic definitions

**DOI:** 10.3389/fcvm.2022.1006104

**Published:** 2022-11-23

**Authors:** T. Craig Cheetham, Susan M. Shortreed, Lyndsay A. Avalos, Kristi Reynolds, Victoria L. Holt, Thomas R. Easterling, Cecilia Portugal, Hui Zhou, Romain S. Neugebauer, Zoe Bider, Abisola Idu, Sascha Dublin

**Affiliations:** ^1^School of Pharmacy, Chapman University, Irvine, CA, United States; ^2^Kaiser Permanente Washington Health Research Institute, Seattle, WA, United States; ^3^Kaiser Permanente Northern California Division of Research, Oakland, CA, United States; ^4^Kaiser Permanente Southern California Department of Research and Evaluation, Pasadena, CA, United States; ^5^Department of Epidemiology, School of Public Health, University of Washington, Seattle, WA, United States; ^6^Department of Obstetrics and Gynecology, University of Washington, Seattle, WA, United States

**Keywords:** pregnancy, hypertension (chronic and gestational), blood pressures, small for gestational age, preterm delivery

## Abstract

**Introduction:**

Studies of hypertension in pregnancy that use electronic health care data generally identify hypertension using hospital diagnosis codes alone. We sought to compare results from this approach to an approach that included diagnosis codes, antihypertensive medications and blood pressure (BP) values.

**Materials and methods:**

We conducted a retrospective cohort study of 1,45,739 pregnancies from 2009 to 2014 within an integrated healthcare system. Hypertensive pregnancies were identified using the “BP-Inclusive Definition” if at least one of three criteria were met: (1) two elevated outpatient BPs, (2) antihypertensive medication fill plus an outpatient hypertension diagnosis, or (3) hospital discharge diagnosis for preeclampsia or eclampsia. The “Traditional Definition” considered only delivery hospitalization discharge diagnoses. Outcome event analyses compared rates of preterm delivery and small for gestational age (SGA) between the two definitions.

**Results:**

The BP-Inclusive Definition identified 14,225 (9.8%) hypertensive pregnancies while the Traditional Definition identified 13,637 (9.4%); 10,809 women met both definitions. Preterm delivery occurred in 20.9% of BP-Inclusive Definition pregnancies, 21.8% of Traditional Definition pregnancies and 6.6% of non-hypertensive pregnancies; for SGA the numbers were 15.6, 16.3, and 8.6%, respectively (*p* < 0.001 for all events compared to non-hypertensive pregnancies). Analyses in women meeting only one hypertension definition (21–24% of positive cases) found much lower rates of both preterm delivery and SGA.

**Conclusion:**

Prevalence of hypertension in pregnancy was similar between the two study definitions. However, a substantial number of women met only one of the study definitions. Women who met only one of the hypertension definitions had much lower rates of adverse neonatal events than women meeting both definitions.

## Introduction

Hypertensive disorders of pregnancy are common and a leading cause of maternal and neonatal morbidity (1). These hypertensive disorders include chronic hypertension, gestational hypertension, preeclampsia superimposed on chronic hypertension, and preeclampsia or eclampsia. Retrospective epidemiologic studies are often used to determine the burden of hypertensive diseases of pregnancy and to evaluate trends over time (2–6).

Studies evaluating the burden of hypertensive disorders of pregnancy generally use discharge diagnosis codes from the delivery hospitalization to estimate overall rates of disease. Diagnosis codes, however, have limitations. Studies evaluating diagnosis codes or a combination of diagnosis codes plus antihypertensive medications report low sensitivity for identifying individuals with hypertension (7, 8). The availability of data from electronic medical records (EMRs) allows for expanding the criteria used to identify and track hypertension in pregnancy. EMRs offer the potential to identify hypertensive disorders of pregnancy using recorded blood pressure (BP) values. This is particularly true in pregnancy because BPs are actively monitored and measured at each prenatal visit.

In a preliminary proof of concept study by Chen et al. we evaluated whether measured and EMR-recorded BP values were useful for identifying hypertension in pregnancy and concluded these BPs were helpful (9). Chen’s study, however, did not require women to be enrolled in the health-plan for the entire pregnancy. In addition, follow-up was censored at 35 weeks 6 days gestation and adverse neonatal outcome events were not assessed (9).

To address these issues, we compared a Traditional Definition for identifying pregnant women with hypertension (using hospital discharge diagnosis codes) to a definition incorporating recorded BPs, plus antihypertensive prescription dispenses and diagnosis codes. Epidemiologically, the objective was to determine the value of a definition including recorded blood compares to the standard definition for identification of hypertensive diseases of pregnancy. For this study we specified that women needed to be enrolled in the health plan for their entire pregnancy and assessed two neonatal outcome events [preterm delivery and small for gestational age (SGA) infants] associated with hypertension (10–17) to evaluate whether the two definitions identified populations of women with similar risk for adverse pregnancy outcomes.

## Materials and methods

### Design and setting

This was a retrospective cohort study set within Kaiser Permanente Southern California (KPSC). KPSC is a large integrated healthcare delivery system providing medical care to over 4.4 million members. Medical care is captured in a comprehensive EMR that includes diagnoses, procedures and treatments from inpatient stays and ambulatory visits, pharmacy dispensing records, vital signs, laboratory results, and radiology reports. These data are linked using a unique medical record number that are retained for life. Pregnancy episodes, mother-infant linkage, and pregnancy specific outcome event data are collected and maintained for research purposes. The institutional review board of KPSC approved the study with a waiver of informed consent.

### Patients

Women who delivered liveborn or stillborn infant(s) from 2009 to 2014 were eligible for inclusion. The delivery year beginning in 2009 was selected because prior to this time, BP measures were not recorded within discrete fields in the EMR. For pregnancy clinical care, KPSC clinicians use self-reported last menstrual period along with first trimester ultrasound data to determine an estimated delivery date (EDD). The start of pregnancy and gestational age were assigned using the EDD established in the EMR.

We included pregnant women between the ages of 15–49 years on the day of delivery who were continuously enrolled in the health-plan from 6 months prior to the start of pregnancy through delivery. Women could contribute more than one pregnancy during the study period. We also required gestational age at birth to be between 22 and 43 weeks because live births outside these gestational ages are implausible. Pregnancies meeting these criteria were included in the base cohort used for descriptive comparisons between study definitions. For the singleton cohort, multiple gestation pregnancies were excluded because of their association with hypertensive disorders of pregnancy and the neonatal outcome events under study.

### Hypertension definitions

Under the ACOG classification of hypertensive disorders of pregnancy, the threshold BP for defining chronic and gestational hypertension is a systolic BP greater than or equal to 140 and/or a diastolic BP greater than or equal to 90 on two occasions at least 4 h apart (18, 19). Gestational hypertension is generally considered to occur after 20 weeks’ gestation. Preeclampsia or eclampsia also occurs after 20 weeks’ gestation and can be superimposed on chronic hypertension. Preeclampsia/eclampsia can also occur after the development of gestational hypertension or can be the presenting hypertensive disorder (18, 19).

The “BP-Inclusive Definition” for identifying hypertensive disorders of pregnancy used three criteria. Hypertension was considered to be present if any of the following criteria were met: (1) two elevated outpatient BPs (systolic BP ≥ 140 and/or diastolic BP ≥ 90) occurring on different days within 30 days of each other from the start of pregnancy through delivery, (2) one or more fills for an antihypertension medication plus one or more hypertension diagnosis codes from the start of pregnancy through delivery (excluding the delivery hospitalization), or (3) one or more hospital discharge diagnosis codes for preeclampsia or eclampsia occurring after 20 weeks’ gestation (see Supplementary Table 1 for a list of diagnosis codes).

The comparison (Traditional) definition for identifying hypertensive disorders of pregnancy used diagnosis codes for chronic hypertension, gestational hypertension, and preeclampsia or eclampsia recorded in the EMR from the delivery hospitalization. As previously noted, this “Traditional Definition” has been used in previous epidemiologic studies evaluating hypertensive disorders in pregnancy (Supplementary Table 1).

Women identified using the BP-Inclusive Definition were classified as having chronic hypertension if they met criteria for hypertension prior to 20 weeks gestation and as having gestational hypertension if they did not meet criteria before 20 weeks but did meet criteria after 20 weeks gestation. A woman was classified as having preeclampsia superimposed on chronic hypertension if she was identified as having chronic hypertension and then went on to have a diagnosis of preeclampsia in the EMR or if she received a hospital diagnosis for this condition after 20 weeks gestation. Lastly, women could be classified as preeclampsia or eclampsia based solely on a hospital diagnosis code after 20 weeks gestation. For women identified using the Traditional Definition, their categorization into these four subgroups was based entirely on delivery hospitalization diagnosis codes found in the EMR. Within KPSC hospital diagnosis codes are entered into the EMR by professional coders based on physician admission notes and discharge summaries. Hospital codes were not validated by chart review for this study.

### Neonatal events

Two neonatal outcome events were evaluated: (1) preterm birth, defined as a delivery prior to 37 weeks 0 days gestational age and (2) SGA, defined as a birthweight less than the 10th percentile based on gender, race and gestational age using published growth curves (20).

### Statistical analysis

The prevalence of hypertensive disorders in pregnancy, overall and by hypertension category (chronic, gestational, etc.,) were determined using the *base cohort* (all pregnancies meeting inclusion criteria between 2009 and 2014). Descriptive comparisons between pregnancies meeting the BP-Inclusive Definition, the Traditional Definition and non-hypertensive pregnancies were conducted using the *singleton cohort* (the base cohort excluding multiple gestation pregnancies). The prevalence of neonatal outcome events were compared between each definition group and the non-hypertensive group from the singleton cohort. Comparisons between these groups were made using Poisson regression with robust variance and *p*-values < 0.05 indicating statistical significance.

Secondary analyses were conducted to assess differences between pregnancies that met the BP-Inclusive Definition and pregnancies that met the Traditional Definition as these two groups are not mutually exclusive. The secondary analyses separated pregnancies into three mutually exclusive groups: (1) those pregnancies who met both hypertension definitions (BP-Inclusive and Traditional Definitions), (2) those who met the study BP-Inclusive Definition but not the Traditional Definition (BP-Inclusive Definition Only), and (3) those who met the Traditional Definition but not the BP-Inclusive Definition (Traditional Definition Only). Again, comparisons between groups were made using Poisson regression with robust variance and *p*-values < 0.05 indicating statistical significance. All analyses were conducted using SAS (SAS Enterprise Guide 7.1; SAS Institute Inc).

## Results

**FIGURE 1:**
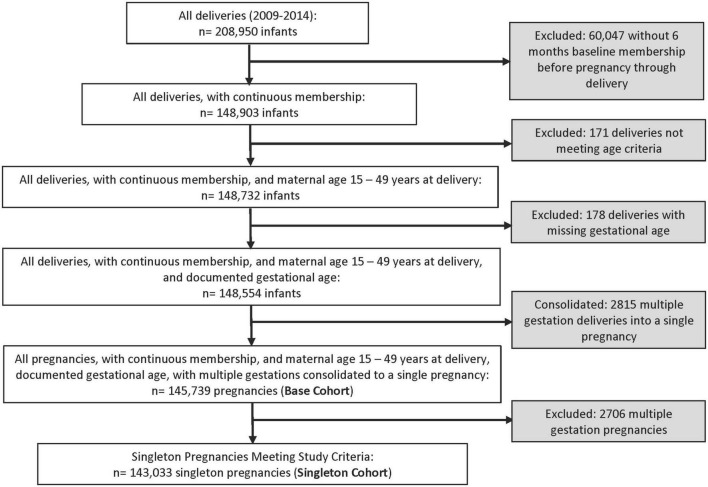
Patient disposition.

The Base Cohort, after applying inclusions and exclusions, consisted of 1,45,739 pregnancies (Figure 1). Most pregnancies (1,28,686 or 88.3%) did not meet either hypertension definition. The prevalence of hypertension was similar using the two definitions; 14,225 (9.8%) met the BP-Inclusive Definition and 13,637 (9.4%) pregnancies met the Traditional Definition. There was considerable overlap between the two study definitions with 10,809 pregnancies meeting both hypertension definitions. However, 24.0% of pregnancies (3,416 of 14,225) met the BP-Inclusive Definition Only and 20.7% (2,828 of 13,637) met the Traditional Definition Only (Table 1). The majority of women (94.2%) who met the BP-Inclusive Definition Only were identified based on the two elevated outpatient BP criteria without a diagnosis at delivery or a diagnosis plus prescription during pregnancy. By comparison, 66.8% of women who met the Traditional Definition Only did not have an outpatient hypertension diagnosis or antihypertensive medication dispensed during pregnancy (Table 1).

**TABLE 1:** Hypertension criteria for non-overlapping hypertensive pregnancies.

Pregnancy met Traditional Definition only	*N* = 2,828
● Antihypertensive drug dispensed but no diagnosis during pregnancy	55 (1.9%)
● Diagnosis but no antihypertensive drug dispensed during pregnancy	885 (31.3%)
● No drug dispensed or diagnosis during pregnancy, only a discharge code	1,888 (66.8%)

**Pregnancy met BP-Inclusive Definition only**	***N*** ** = 3,416**

● Met 2 blood pressure and diagnosis plus prescription dispense criteria	96 (2.8%)
● Met diagnosis plus prescription dispense criteria only	101 (3.0%)
● Met 2 blood pressure criteria only	3,219 (94.2%)

For the individual criteria used in the BP-Inclusive Definition: 7,990 (56.2%) pregnancies met the two elevated BP criteria, 3,166 (22.3%) pregnancies met the diagnosis plus prescription criteria and 7,598 (53.4%) pregnancies had a hospital diagnosis of preeclampsia/eclampsia. Pregnancies could meet more than one criterion. Additional details regarding the base cohort are provided in Table 2 (Hypertensive Disorders of Pregnancy in women meeting the BP Inclusive Definition) and Supplementary Table 2 (Demographic Characteristics of the Base Cohort).

**TABLE 2:** Breakdown of hypertensive disorders of pregnancy in the base cohort for 14,225 women identified using the BP-Inclusive Definition.

Hypertensive disorders of pregnancy	Percent (N)
**Chronic hypertension (*n* = 4,276)**	
Chronic hypertension	22.3% (3,170)
Chronic hypertension who went on to develop preeclampsia	7.8% (1,106)
**Gestational hypertension (*n* = 4,776)**	
Gestational hypertension	24.3% (3,457)
Gestational hypertension who went on to develop preeclampsia	9.3% (1,319)
**Preeclampsia-eclampsia** (without evidence of chronic or gestational hypertension)	36.4% (5,173)
Total	100% (14,225)

Demographic information for the singleton cohort by hypertension definition is reported in Table 3. The average age for women with a hypertensive disorder of pregnancy (using either definition) was higher than the age for the non-hypertensive pregnancies, driven primarily by a higher percentage of hypertensive women in the 35–50 years age group. Women whose pregnancy met one of the hypertension definitions were also more likely to be obese (46.1–48.5% versus 23%), to be nulliparous (44.7–44.8% versus 40.1%) and to have co-morbid conditions (diabetes, heart disease or renal disease) than non-hypertensive women. These finding were consistent for both hypertension definitions.

**TABLE 3:** Baseline characteristics for the singleton cohort by hypertension definition (2009–2014).

Characteristic	Non-hypertensive (*n* = 126,682)	Traditional Definition* (*n* = 13,033)	BP-Inclusive Definition** (*n* = 13,592)
**Maternal age at delivery,** Mean ± SD	29.9 ± 5.8	31.0 ± 6.3	31.0 ± 6.2
**Age at delivery, *N* (%)**			
15–19	6,462 (5.1)	599 (4.6)	562 (4.1)
20–24	17,111 (13.5)	1,603 (12.3)	1,645 (12.1)
25–29	33,187 (26.2)	2,824 (21.7)	3,064 (22.5)
30–34	41,967 (33.1)	4,010 (30.8)	4,205 (30.9)
35–50	27,955 (22.1)	3,997 (30.7)	4,116 (30.3)
**Race/Ethnicity, *N* (%)**			
White	32,232 (25.4)	3,080 (23.6)	3,628 (26.7)
Asian	16,622 (13.1)	1,416 (10.9)	1,444 (10.6)
Black	9,831 (7.8)	1,692 (13)	1,659 (12.2)
Hispanic	65,772 (51.9)	6,566 (50.4)	6,582 (48.4)
Other	2,225 (1.8)	279 (2.1)	279 (2.1)
**Maternal education, *N* (%)**			
Less than high school	10,377 (8.2)	1,065 (8.2)	1,030 (7.6)
High school	29,770 (23.5)	3,043 (23.3)	3,201 (23.6)
College	67,497 (53.3)	7,187 (55.1)	7,541 (55.5)
Graduate	18,840 (14.9)	1,724 (13.2)	1,806 (13.3)
Unknown	198 (0.2)	14 (0.1)	14 (0.1)
**BMI, *N* (%)**			
<18.5	8,504 (6.7)	658 (5)	616 (4.5)
18.5–24.9	54,535 (43)	2,952 (22.7)	2,940 (21.6)
25.0–29.9	34,467 (27.2)	3,414 (26.2)	3,444 (25.3)
>30.0	29,174 (23)	6,009 (46.1)	6,591 (48.5)
Missing	2 (0)	0	1 (0)
**Parity, *N* (%)**			
0	50,824 (40.1)	5,845 (44.8)	6,073 (44.7)
1	40,124 (31.7)	3,662 (28.1)	3,829 (28.2)
2	17,268 (13.6)	1,586 (12.2)	1,677 (12.3)
>3	8,443 (6.7)	832 (6.4)	864 (6.4)
Missing	10,023 (7.9)	1,108 (8.5)	1,149 (8.5)
**Co-morbidities, *N* (%)**			
Diabetes	1,051 (0.8)	713 (5.5)	715 (5.3)
Heart disease	311 (0.2)	61 (0.5)	57 (0.4)
Renal disease	87 (0.1)	91 (0.7)	92 (0.7)
**Outpatient blood pressures, median, (IQR)**			
Total blood pressures	14 (11, 17)	19 (14, 28)	18 (14, 28)
Total elevated blood pressures	–	4 (2, 7)	3 (2, 7)
Time between first and last elevated blood pressure, days	–	126 (25, 197)	152 (52, 203)

*“Traditional Definition” is based on discharge diagnosis codes from the delivery hospitalization. **“BP-Inclusive Definition” is based on measured blood pressure values, diagnosis codes and dispensed antihypertensive medications. 10,809 women met both the Traditional and the BP-Inclusive Definition.

Over 2.24 million outpatient BPs were recorded for the 1,43,033 pregnancies in the singleton cohort. The median number of outpatient BPs recorded during pregnancy in the non-hypertensive cohort was 14 while the median number of BPs ranged between 18 and 19 in pregnancies meeting one of the hypertension definitions (Table 3). As expected, pregnancies meeting the hypertension definitions had more documented elevated BPs than the non-hypertensive pregnancies; pregnancies meeting either one of the two hypertensive definitions had a median of three or four elevated BPs recorded over a 4-to-5-month period of time (126–152 days).

The BP-Inclusive Definition identified more deliveries as having chronic hypertension (22.7%) than the Traditional Definition (18.4%) (Table 4). Preeclampsia superimposed on chronic hypertension was also higher in the BP-Inclusive Definition cohort (10.6% versus 8.3%) for similar reasons; a higher percentage identified with chronic hypertension led to more women being classified as having preeclampsia superimposed on chronic hypertension. The proportion of women with just preeclampsia or eclampsia was lower in pregnancies meeting the BP-Inclusive Definition compared to the Traditional Definition (42.0 versus 46.6%, respectively).

**TABLE 4:** Hypertension subgroups by hypertension definition (singleton cohort).

Hypertension subgroup	Traditional Definition % (*N* = 13,033)	BP-Inclusive Definition % (*N* = 13,592)
Chronic hypertension	18.4% (2,400)	22.7% (3,078)
Gestational hypertension	26.7% (3,483)	24.8% (3,364)
Preeclampsia superimposed on chronic hypertension	8.3% (1,083)	10.6% (1,441)
Preeclampsia/Eclampsia	46.6% (6,067)	42.0% (5,709)

**FIGURE 2:**
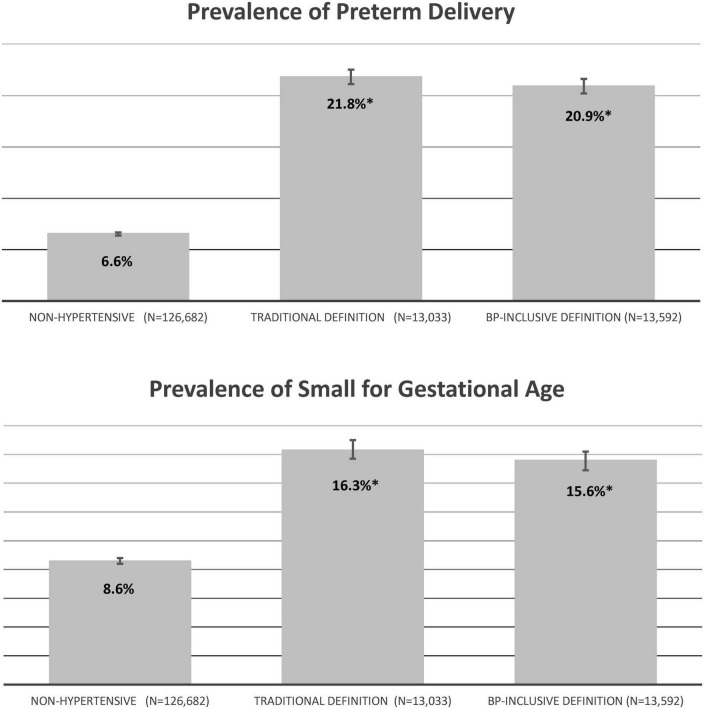
Pregnancy outcomes for the singleton cohort by hypertension definition (percent). Non-hypertensive – pregnancies that did not meet either hypertension definition; Traditional Definition – pregnancies who met the Traditional Definition based on delivery hospitalization discharge codes as having hypertension; BP-Inclusive Definition – pregnancies who met the BP-Inclusive Definition based on elevated blood pressures, prescriptions and a diagnosis or delivery diagnoses for eclampsia/preeclampsia as having hypertension. *P < 0.001 versus non-hypertensive pregnancies.

The prevalence of preterm delivery and SGA infants were similar for pregnancies meeting the BP-Inclusive Definition and the Traditional Definition and were significantly higher than for women without evidence of hypertensive disorders of pregnancy (Figure 2).

**FIGURE 3:**
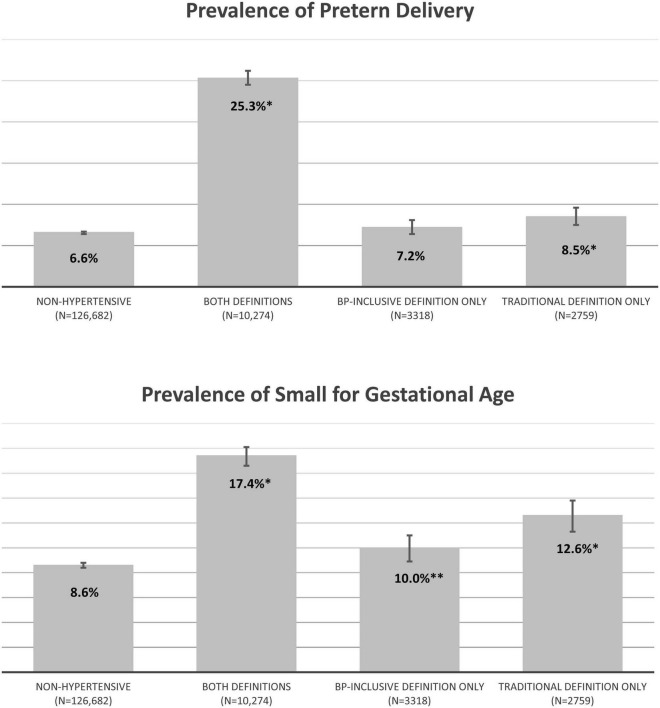
Secondary analysis of pregnancy outcomes for the singleton cohort by mutually exclusive hypertension groups (percent). Non-hypertensive – pregnancies that did not meet either hypertension definition; Both Definitions – pregnancies that were identified as hypertensive by both study definitions; Traditional Definition Only – pregnancies that met the Traditional Definition but not the BP-Inclusive Definition; BP-Inclusive Definition Only – pregnancies that met the BP-Inclusive Definition but not the Traditional Definition. *P < 0.001 compared to non-hypertensive pregnancies. **P = 0.006 compared to non-hypertensive pregnancies.

The secondary analysis separated hypertensive women into three mutually exclusive groups, the baseline characteristics of these three groups were similar to those found in the base cohort (Supplementary Table 3). The highest prevalence of adverse neonatal outcome events were seen in women who met both study definitions (Preterm Delivery = 25.3%, SGA = 17.4%) (Figure 3). The prevalence of neonatal outcome events for women meeting one of the hypertension definitions but not the other were also higher than the prevalence found for non-hypertensive pregnancy but markedly less elevated. Both outcomes for pregnancies meeting the Traditional Definition Only were statistically higher than non-hypertensive pregnancies (Preterm Delivery 8.5% versus 6.6%; SGA 10.6% versus 8.6%). This, however, was not true for pregnancies meeting the BP-Inclusive Definition Only. For the preterm delivery outcome, pregnancies meeting the BP-Inclusive Definition had a prevalence of 7.2% compared to 6.6% in non-hypertensive pregnancies (*p* = 0.13).

## Discussion

In this study, we compared two epidemiologic definitions for identifying hypertensive disorders of pregnancy. Both approaches identified a similar prevalence of hypertensive disorders, and as expected, hypertensive pregnancies identified using these definitions had an increased prevalence of adverse neonatal outcome events compared to pregnancies not identified as hypertensive. Overall, a high percentage of hypertensive pregnancies met both hypertension definitions but there was a significant percentage of pregnancies who met only one of the two hypertension definitions used in this study (i.e., 21–24% of pregnancies met one of the definitions but not the alternative.).

Secondary analyses focused on the three mutually exclusive populations of hypertensive pregnancies. These analyses found that women who met both hypertension definitions had the highest prevalence of adverse neonatal outcome events. Women who met either the BP-Inclusive Definition Only or the Traditional Definition Only had a lower prevalence of adverse fetal outcomes, although these prevalence’s were still higher than those seen in non-hypertensive women. In pregnancies identified using the BP-Inclusive definition Only (identified based strictly on the elevated BP criterion) the prevalence of SGA was significantly higher than in non-hypertensive pregnancies, while the prevalence of pre-term delivery was not statistically different.

The significance of these findings is unknown and will require additional study. Specifically, for women who met the Traditional Definition Only, it is worth evaluating their clinical profile to understand why they received a hypertension diagnosis at delivery without other evidence of hypertension during pregnancy and why these women had much lower rates of adverse pregnancy outcomes. Often these women receive no antihypertensive medications during pregnancy because US guidelines do not recommend treatment for moderate hypertension in pregnancy. However, a good percentage received a diagnosis of hypertension during pregnancy without meeting the elevated BP criteria specified in the BP-Inclusive Definition. There are several potential explanations. First, hypertensive women may stop their BP medications to prevent potential harmful exposures to the fetus. Second, BPs are known to decline early in pregnancy with a gradual increase in the second and third trimester; it is possible that the increase in BP during the second and third trimester never reached the threshold BP of ≥140/90 in some of these women. Third, we restricted qualifying BPs to those recorded in an outpatient setting; expanding this criterion to include inpatient BPs could increase the number of women meeting the elevated-BP standard. And lastly, some of these women may have had evidence of hypertension only during their delivery hospitalization.

Most of the women who met the BP-Inclusive Definition Only were identified based on the elevated-BP criteria. It is important to understand why these women did not receive a diagnosis of hypertension. Chart reviews were conducted in our prior study to evaluate discrepancies between various hypertension definitions compared to the definition that incorporated BP values (9). Among a sample of women with elevated BP values but without a diagnosis of hypertension, 58% had evidence in the chart that the elevated BPs were recognized by the provider. Reasons for the lack of a hypertension diagnosis in this previous study could not be determined (9). It is possible that providers are reluctant to assign a diagnosis of hypertension if there is no plan for treatment. It is also possible that if multiple BP values were obtained on a single day, including some that were elevated and others that were not; providers may have given more weight to the values that were not elevated. Additional work needs to be done to understand why hypertension diagnoses are not recorded in the EMR of women with elevated BPs.

This study included a large sample of women and over 2.2 million outpatient BP values recorded during pregnancy. If, as previous studies suggest, diagnosis codes for hypertension have low sensitivity (7, 8) then measured BPs have the potential to identify and support the diagnosis of hypertension. The inclusion of measured BPs is attractive from an epidemiologic standpoint because it allows for quantification of risk based on BP level and BP variability during pregnancy (21). However, pregnancies identified based only on the BP criterion used in this study were associated with a very small increase in the prevalence of adverse neonatal outcome events compared to pregnancies with no evidence of hypertension. The time between first and last elevated BP was also shorter in the BP only group, 63 days versus 152 days in the full group meeting the BP-Inclusive Definition. Additionally, women meeting the elevated BP only criterion had a median of 4 high BPs out of 21 total measured during pregnancy. These findings support the need for further work in this area.

Several limitations need to be considered when evaluating the results of this study. First, the elevated BP measures used for the BP-Inclusive Definition may not represent true hypertension; elevated BPs can arise in a variety of clinical circumstances (such as white-coat hypertension, pain or stress related conditions and medications like decongestants or non-steroidal anti-inflammatory drugs). Second, the BP criteria used in the study, two elevated BPs on different days but with 30 days of each other, could be flawed. It could be argued that given the observational nature of these data, a higher number of elevated BPs or a different time interval between measurements are necessary to establish hypertension. Our criteria were designed to capture what might reasonably occur in clinical practice where a woman has elevated outpatient BPs on two separate visits before hypertension is diagnosed; the 30-day time frame was designated so that BP elevations were within a relatively short time span and not spread out over a 280 day pregnancy. Future studies could look at changing the required number of BPs and timing of the BP measures needed to define hypertension from electronic health records. Third, we only evaluated two neonatal outcomes; maternal outcomes that could be studied in the future include maternal intensive care unit admissions and cardiovascular or cerebrovascular outcomes.

## Conclusion

Both epidemiologic definitions used in this study identified similar prevalences of pregnancies complicated by hypertension. A high number of hypertensive pregnant women met criteria for both definitions, however, there was a significant proportion of pregnancies that met one but not both definitions. The prevalence of neonatal outcome events was different between women who met both definitions versus those meeting only a single definition. Additional work needs to be done understand the reasons and importance of these outcome event differences in hypertensive pregnant women meeting different epidemiologic definitions.

## Data availability statement

The datasets presented in this article are not readily available. Anonymized data that support the findings of this study may be made available from the investigative team in the following conditions: (1) agreement to collaborate with the study team on all publications, (2) provision of external funding for administrative and investigator time necessary for this collaboration, (3) demonstration that the external investigative team is qualified and has documented evidence of training for human subjects protections, and (4) agreement to abide by the terms outlined in data use agreements between institutions. Requests to access the datasets should be directed to kristi.reynolds@kp.org.

## Ethics statement

This study involving human participants was reviewed and approved by the Kaiser Permanente Southern California Institutional Review Board. The board waived the requirement for signed informed consent.

## Author contributions

TC, SS, LA, VH, TE, RN, AI, and SD: concept and design of the study. LA, CP, HZ, ZB, and AI: acquisition of study data. SS, VH, HZ, RN, and ZB: analysis of the data. TC, SS, LA, VH, TE, HZ, RN, ZB, AI, and SD: interpretation of the results. TC, SS, VH, and HZ: drafting of the manuscript. TC, SS, LA, KR, VH, TE, CP, HZ, AI, and SD: critical revisions for important intellectual content. All authors contributed to the article and approved the submitted version.
