# Supplementary material for: Identifying hypertensive disorders of pregnancy, a comparison of two epidemiologic definitions
Source: Front Cardiovasc Med. 2022 Nov 23;9:1006104. doi: 10.3389/fcvm.2022.1006104 (PMC9727220; doi:10.3389/fcvm.2022.1006104)
Supplement: Supplementary file 1 [file Data_Sheet_1.doc]

Supplemental Tables and Figures

**Supplemental Table 1 - List of ICD9 Codes and Descriptions for Hypertension**

| **Hypertension Complicating Pregnancy, Childbirth and Puerperium** | | | |
| --- | --- | --- | --- |
| ***Code*** | | ***Description*** | ***Type*** |
| 642.0x | | Benign essential hypertension | Chronic |
| 642.1x | | Hypertension secondary to renal disease | Chronic |
| 642.2x | | Other pre-existing hypertension | Chronic |
| 642.3x | | Transient hypertension of pregnancy (gestational hypertension) | Gestational |
| 642.9x | | Unspecified hypertension of pregnancy | Chronic |
| **Circulatory System – Hypertensive Diseases** | | | |
| ***Code*** | ***Description*** | | ***Type*** |
| 401 | Essential hypertension | | Chronic |
| 401.0 | Malignant essential hypertension | | Chronic |
| 401.1 | Benign essential hypertension | | Chronic |
| 401.9 | Unspecified essential hypertension | | Chronic |
| 402 | Hypertensive Heart Disease | | Chronic |
| 402.0 | Malignant hypertensive heart disease | | Chronic |
| 402.00 | Malignant hypertensive heart disease – without heart failure | | Chronic |
| 402.01 | Malignant hypertensive heart disease – with heart failure | | Chronic |
| 402.1 | Benign hypertensive heart disease | | Chronic |
| 402.10 | Benign hypertensive heart disease – without heart failure | | Chronic |
| 402.11 | Benign hypertensive heart disease – with heart failure | | Chronic |
| 403 | Hypertensive chronic kidney disease | | Chronic |
| 403.0x | Malignant hypertensive chronic kidney disease | | Chronic |
| 403.1x | Benign hypertensive chronic kidney disease | | Chronic |
| 403.9x | Unspecified hypertensive chronic kidney disease | | Chronic |
| 404 | Hypertensive heart and chronic kidney disease | | Chronic |
| 404.0x | Malignant hypertensive heart and chronic kidney disease | | Chronic |
| 404.1x | Benign hypertensive heart and chronic kidney disease | | Chronic |
| 404.9x | Unspecified hypertensive heart and chronic kidney disease | | Chronic |
| 405 | Secondary hypertension | | Chronic |
| 405.0 | Malignant secondary hypertension | | Chronic |
| 405.01 | Malignant renovascular secondary hypertension | | Chronic |
| 405.09 | Other malignant secondary hypertension | | Chronic |
| 405.1 | Benign secondary hypertension | | Chronic |
| 405.11 | Benign renovascular secondary hypertension | | Chronic |
| 405.19 | Other benign secondary hypertension | | Chronic |
| 405.9 | Unspecified secondary hypertension | | Chronic |
| 405.91 | Unspecified renovascular secondary hypertension | | Chronic |
| 405.99 | Other unspecified renovascular secondary hypertension | | Chronic |
| **Preeclampsia & Eclampsia** | | | |
| ***Code*** | ***Description*** | | ***Type*** |
| 642.4x | Mild or unspecified preeclampsia | | Preeclampsia |
| 642.5x | Severe preeclampsia | | Preeclampsia |
| 642.6x | Eclampsia | | Eclampsia |
| 642.7x | Preeclampsia or eclampsia superimposed on pre-existing hypertension | | Preeclampsia superimposed on chronic hypertension |

**Supplemental Table 2: Baseline Characteristics of the Base Cohort by Hypertensive Definition (2009-2014)**

| ***Characteristic*** | *Non-Hypertensive* | *Traditional Definition* | *BP-Inclusive Definition* |
| --- | --- | --- | --- |
|  | *(n=*128,686*)* | *(n=13,637)* | *(n=14,225)* |
| **Maternal age at delivery, mean ± SD** | 29.9 ± 5.8 | 31.0 ± 6.3 | 31.1 ± 6.2 |
| **Age at delivery, N (%)** |  |  |  |
| 15-19 | 6503 (5.1) | 614 (4.5) | 579 (4.1) |
| 20-24 | 17277 (13.4) | 1646 (12.1) | 1688 (11.9) |
| 25-29 | 33638 (26.1) | 2928 (21.5) | 3179 (22.3) |
| 30-34 | 42698 (33.2) | 4220 (30.9) | 4428 (31.1) |
| 35-50 | 28570 (22.2) | 4229 (31) | 4351 (30.6) |
| **Race/Ethnicity, N (%)** |  |  |  |
| White | 32899 (25.6) | 3262 (23.9) | 3834 (27) |
| Asian | 16914 (13.1) | 1498 (11) | 1521 (10.7) |
| Black | 10008 (7.8) | 1771 (13) | 1740 (12.2) |
| Hispanic | 66608 (51.8) | 6817 (50) | 6838 (48.1) |
| Other | 2257 (1.8) | 289 (2.1) | 292 (2.1) |
| **BMI, N (%)** |  |  |  |
| < 18.5 | 8596 (6.7) | 681 (5) | 637 (4.5) |
| 18.5-24.9 | 55419 (43.1) | 3168 (23.2) | 3144 (22.1) |
| 25.0-29.9 | 35014 (27.2) | 3554 (26.1) | 3592 (25.3) |
| >30.0 | 29655 (23) | 6234 (45.7) | 6851 (48.2) |
| Missing | 2 (0) |  | 1 (0) |
| **Multiple birth, N (%)** | 2004 (1.6) | 604 (4.4) | 633 (4.4) |
| **Parity, N (%)** |  |  |  |
| 0 | 51847 (40.3) | 6192 (45.4) | 6428 (45.2) |
| 1 | 40636 (31.6) | 3793 (27.8) | 3969 (27.9) |
| 2 | 17483 (13.6) | 1634 (12) | 1725 (12.1) |
| >3 | 8565 (6.7) | 864 (6.3) | 903 (6.3) |
| Missing | 10155 (7.9) | 1154 (8.5) | 1200 (8.4) |
| **Co-morbidities, N (%)** |  |  |  |
| Diabetes | 1072 (0.8) | 731 (5.4) | 733 (5.2) |
| Heart disease | 316 (0.2) | 62 (0.5) | 58 (0.4) |
| Renal disease | 88 (0.1) | 92 (0.7) | 93 (0.7) |
| **Blood Pressures** (median, IQR) |  |  |  |
| Total Blood Pressures | 14  (11.0, 17.0) | 18  (13.0, 27.0) | 20  (14.0, 28.0) |
| Total Elevated Blood Pressures | -- | 3  (2.0, 7.0) | 4  (2.0, 7.0) |
| Time Between First and Last Elevated Blood Pressure, Days | -- | 150  (51.0, 202.0) | 126  (25.0, 197.0) |

**Supplemental Table 3: Characteristics of the Analysis Cohort by Subgroups for the Secondary Analysis Categories (2009-2014)**

| ***Baseline Characteristic*** | **Non-Hypertensive Cohort*** | **Women Meeting Both Hypertension Definitions*** | **Women Meeting the BP-Inclusive Definition but Not Traditional Definition*** | **Women Meeting the Traditional Definition but Not BP-Inclusive Definition*** |
| --- | --- | --- | --- | --- |
|  | **(n=126,682)** | **(n=10,274)** | **(n=3,318)** | **(n=2,759)** |
| **Maternal age at delivery, mean ±SD** | 29.9 ± 5.8 | 31.1 ± 6.3 | 30.6 ± 5.9 | 30.4 ± 6.2 |
| **Age at delivery, N (%)** |  |  |  |  |
| 15-19 | 6462 (5.1) | 459 (4.5) | 103 (3.1) | 140 (5.1) |
| 20-24 | 17111 (13.5) | 1226 (11.9) | 419 (12.6) | 377 (13.7) |
| 25-29 | 33187 (26.2) | 2195 (21.4) | 869 (26.2) | 629 (22.8) |
| 30-34 | 41967 (33.1) | 3137 (30.5) | 1068 (32.2) | 873 (31.6) |
| 35-50 | 27955 (22.1) | 3257 (31.7) | 859 (25.9) | 740 (26.8) |
| **Race/Ethnicity, N (%)** |  |  |  |  |
| White | 32232 (25.4) | 2350 (22.9) | 1278 (38.5) | 730 (26.5) |
| Asian | 16622 (13.1) | 1154 (11.2) | 290 (8.7) | 262 (9.5) |
| Black | 9831 (7.8) | 1343 (13.1) | 316 (9.5) | 349 (12.6) |
| Hispanic | 65772 (51.9) | 5203 (50.6) | 1379 (41.6) | 1363 (49.4) |
| Other | 2225 (1.8) | 224 (2.2) | 55 (1.7) | 55 (2) |
| **Maternal education, N (%)** |  |  |  |  |
| Less than High school | 10377 (8.2) | 859 (8.4) | 171 (5.2) | 206 (7.5) |
| High school | 29770 (23.5) | 2366 (23) | 835 (25.2) | 677 (24.5) |
| College | 67497 (53.3) | 5708 (55.6) | 1833 (55.2) | 1479 (53.6) |
| Graduate | 18840 (14.9) | 1333 (13) | 473 (14.3) | 391 (14.2) |
| Unknown | 198 (0.2) | 8 (0.1) | 6 (0.2) | 6 (0.2) |
| **BMI, N (%)** |  |  |  |  |
| < 18.5 | 8504 (6.7) | 502 (4.9) | 114 (3.4) | 156 (5.7) |
| 18.5-24.9 | 54535 (43) | 2229 (21.7) | 711 (21.4) | 723 (26.2) |
| 25.0-29.9 | 34467 (27.2) | 2628 (25.6) | 816 (24.6) | 786 (28.5) |
| >30.0 | 29174 (23) | 4915 (47.8) | 1676 (50.5) | 1094 (39.7) |
| Missing | 2 (0) |  | 1 (0) |  |
| **Parity, N (%)** |  |  |  |  |
| 0 | 50824 (40.1) | 4580 (44.6) | 1493 (45) | 1265 (45.8) |
| 1 | 40124 (31.7) | 2868 (27.9) | 961 (29) | 794 (28.8) |
| 2 | 17268 (13.6) | 1278 (12.4) | 399 (12) | 308 (11.2) |
| >3 | 8443 (6.7) | 671 (6.5) | 193 (5.8) | 161 (5.8) |
| Missing | 10023 (7.9) | 877 (8.5) | 272 (8.2) | 231 (8.4) |
| **Co-morbidities, N (%)** |  |  |  |  |
| Diabetes | 1051 (0.8) | 619 (6) | 96 (2.9) | 94 (3.4) |
| Heart disease | 311 (0.2) | 48 (0.5) | 9 (0.3) | 13 (0.5) |
| Renal disease | 87 (0.1) | 80 (0.8) | 12 (0.4) | 11 (0.4) |
| **Number of Blood Pressure** **measured from start of pregnancy to delivery, Median (IQR)** |  |  |  |  |
| Total Blood Pressures (median, IQR) | 14  (11, 17) | 19  (13, 28) | 21  (16, 28) | 16  (12, 21) |
| Total Elevated Blood Pressures (median, IQR) | 1  (1, 2) | 4  (2, 8) | 4  (2, 5) | 1  (1, 2) |
| Time Between First and Last Elevated Blood Pressure (median, IQR) | 134  (75, 191) | 151  (48, 203) | 63  (16, 180) | 153  (85, 197) |

* These groups are mutually exclusive
